# SERUM VALUES OF ALKALINE PHOSPHATASE AND LACTATE DEHYDROGENASE IN OSTEOSARCOMA

**DOI:** 10.1590/1413-785220162403157033

**Published:** 2016

**Authors:** JUAN PABLO ZUMÁRRAGA, ANDRÉ MATHIAS BAPTISTA, LUIS PABLO DE LA ROSA, MARCELO TADEU CAIERO, OLAVO PIRES DE CAMARGO

**Affiliations:** 1. Universidade de São Paulo, Faculdade de Medicina, Hospital das Clínicas, Instituto de Ortopedia e Traumatologia, São Paulo, SP, Brazil.; 2. Universidade de São Paulo, Faculdade de Medicina, Department of Orthopedics and Traumatology, São Paulo, SP, Brazil.

**Keywords:** Osteosarcoma, Alkaline phosphatase, L-Lactate Dehydrogenase, Tumor necrosis factors, Drug therapy, Prognosis.

## Abstract

**Objective:**

To study the relationship between the pre and post chemotherapy (CT) serum levels of alkaline phosphatase (AP) and lactate dehydrogenase (LDH), and the percentage of tumor necrosis (TN) found in specimens after the pre surgical CT in patients with osteosarcoma.

**Methods:**

Series of cases with retrospective evaluation of patients diagnosed with osteosarcoma. Participants were divided into two groups according to serum values of both enzymes. The values of AP and LDH were obtained before and after preoperative CT. The percentage of tumor necrosis (TN) of surgical specimens of each patient was also included.

**Results:**

One hundred and thirty seven medical records were included from 1990 to 2013. Both the AP as LDH decreased in the patients studied, being the higher in pre CT than post CT. The average LHD decrease was 795.12U/L and AP decrease was 437.40 U/L. The average TN was 34.10 %. There was no statistically significant correlation between the serums values and the percentage of tumoral necrosis.

**Conclusion:**

The serum levels values of AP and LDH are not good predictors for the chemotherapy-induced necrosis in patients with osteosarcoma. ***Level of Evidence IV, Case Series.***

## INTRODUCTION 

Osteosarcoma (OS) is the most common among primary tumors of non-hematopoietic bone. With an estimated incidence of five cases per million, there is no significant association with ethnic or racial groups. It is especially common in young people. If no treatment is offered, the disease is invariably fatal, due to its local aggressive behavior and spread, mainly for the lung.^1^ ^-^ ^7^

The survival rate has improved considerably with the introduction and evolution of chemotherapy. However, the identification of prognostic factors for osteosarcoma has been a major problem for Orthopedic Oncology centers. The age, gender, anatomical location, tumor size and serum values of Alkaline Phosphatase (AF) and Lactate Dehydrogenase (LDH) and percentage of necrosis are some of the prognostic factors that have been studied in osteosarcoma.^8^ ^-^ ^10^

The osteosarcoma response to chemotherapy is considered one of the most important indicators of global patient survival. This response is measured by the percentage of necrosis found in the resected tumor reported by the pathologist.^11^ ^-^ ^15^

## MATERIALS AND METHODS

The study was approved by the Ethics Committee of the Department of Orthopedics and Traumatology, IOTHC-USP under number 8677/2013. It is a retrospective study and the authors used the medical records of patients diagnosed with osteosarcoma who were treated by the Orthopedic Oncology Group of the Institute of Orthopedics and Traumatology, *Hospital das Cl*í*nicas, Faculdade de Medicina da Universidade de São Paulo* (IOTHC-USP), in the period from 1990 to September 2013.

Patients diagnosed with osteosarcoma by pathological studies, whose records of serum AP and LDH before and after preoperative chemotherapy and post-chemotherapy necrosis data were available, were included in the study.

We studied 647 medical records of patients with pathological diagnosis of OS. We obtained from medical records the following epidemiological information: gender, anatomical location of the tumor, histological diagnosis and the patient's age. We also collected serum values of AP and LDH before and after preoperative chemotherapy, as well as the percentage of tumor necrosis from each resected specimen. Of the 647 original records, 510 were excluded because they did not have complete data for further analysis. The study included, thus, 137 records. The serum values of AP and LDH of the patients included were obtained before and after preoperative chemotherapy. The percentage of tumor necrosis of each resected specimen was also collected.

Regarding the study sample, the mean age of patients was 23.0 ± 14.5 years old. Most patients (65.9%) were male, and 34.1% were female. The most frequent tumor location was the femur (56.5%), followed by the tibia (18.8%). We classified AP and LDH results into two groups: Group 1, formed by patients with normal values of AP and LDH, according to the reference values of HCFMUSP laboratory (LDH range 24 - 480 U/L, AP range 35 - 130 U/L). Analyses were performed by a Roche/Hitachi COBAS modular machine. Group 2 was formed by patients with AP and LDH values above the normal range. The classification used to gather serologic values under study was adapted from Bramer et al.^16^ The percentages of tumor necrosis described by pathologists in specimens extracted during surgery, after chemotherapy, were also recorded. These percentages were used according to the rate of tumor necrosis described by Huvos.^11^ Data were expressed as mean and standard deviation.

### Statistical analysis

In order to verify the correlation between the percentage of tumor necrosis and serum values of enzymes, pre- and post-chemotherapy, or a variation thereof, the following statistical analyzes were made: the nominal characteristics of patients with relative and absolute frequencies were described. The patients' age ranges were described as mean and standard deviation.^17^

The values of AP and LDH and its variations with chemotherapy, as well as the percentage of tumor necrosis were described with summary measurements (mean, standard deviation, median, minimum and maximum). The correlation between necrosis of the surgical specimen and serum values of enzymes pre and post chemotherapy and the difference (post minus pre) and also between enzymes were calculated and verified by Spearman correlation. The tests were performed at 5% significance level. The software used for the statistical analysis were SPSS 20.0 (Statistical Program for Social Sciences version 20.0 for Windows) and Microsoft Excel 2008.

## RESULTS

Both AP as LDH decreased among our patients comparing pre- and post-chemotherapy values. The mean difference of pre- and post-chemotherapy AP was 795.12 mg/dL, and the mean difference of pre- and post-chemotherapy LDH was 437.40 U/L. The mean tumor necrosis percentage was 34.10 ± 24.09. The values ranged between 0% and 100% tumor necrosis, respectively. (Table 1)

**Table 1:**
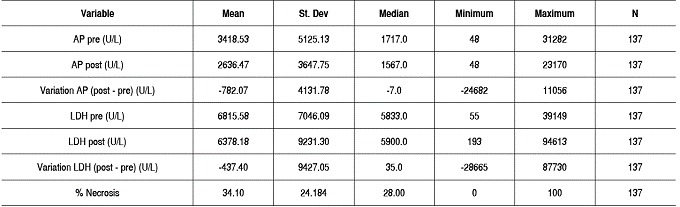
Description of enzymes, variation with chemotherapy and percentage of necrosis of surgical specimens.

AP: Alkaline Phosphatase; LDH: Lactate Dehydrogenase; St.Dev: Standard Deviation; N: number.

**Figure 1:**
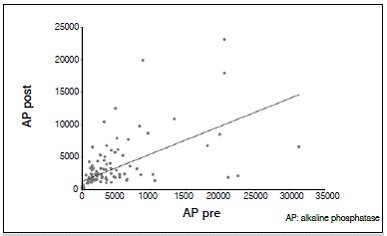
Dispersion diagram of AP pre- and post-chemotherapy.

There was no statistically significant correlation between tumor necrosis and AP and LDH values. The lack of correlation was observed with both pre- and post-chemotherapy values, and there was no correlation of enzymes levels after chemotherapy (*p* <0.05). For AP and LDH there was a direct correlation at each time point, as well as the relationship between values pre- and post-chemotherapy for each enzyme (*p* <0.05). These results are shown in Table 2 and Figures 1-^6^.

**Table 2:**
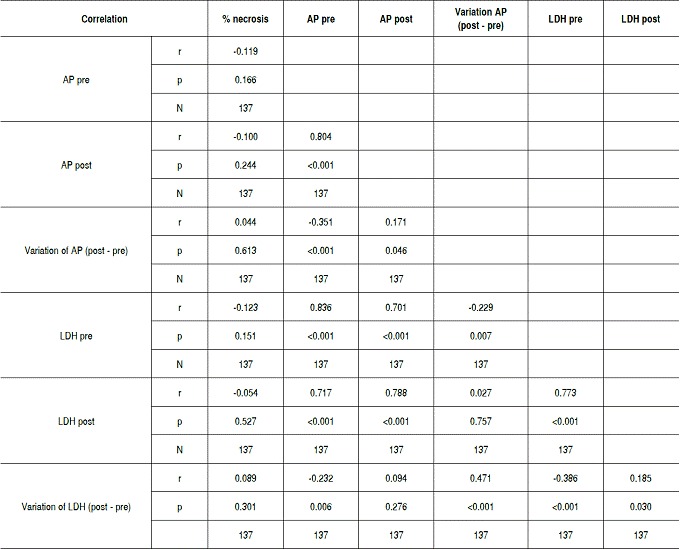
Result of correlation of percentage of necrosis with AP and LDH values, and between both enzymes at each moment of assessment and their variation post-chemotherapy.

AP: Alkaline Phosphatase; LDH: Lactate Dehydrogenase; N: number.

**Figure 2:**
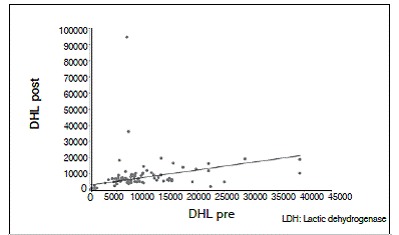
Dispersion diagram of LDH pre- and post-chemotherapy.

**Figure 3:**
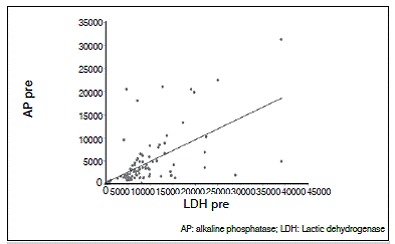
Dispersion diagram of LDH and AP pre-chemotherapy.

**Figure 4:**
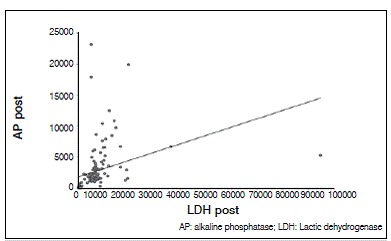
Dispersion diagram of LDH and AP post-chemotherapy.

**Figure 5:**
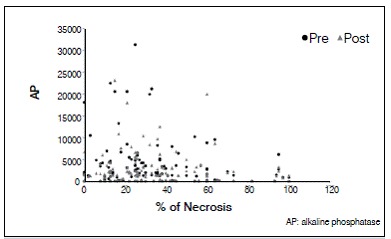
Dispersion diagram of percentage of necrosis and AP pre- and post chemotherapy.

**Figure 6:**
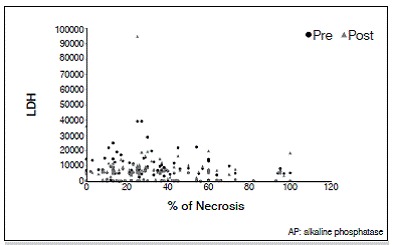
Dispersion diagram of percentage of necrosis and LDH pre- and post chemotherapy.

## DISCUSSION

One of the main osteosarcoma prognostic factors is tumor necrosis found in the surgical specimen. However, this information can only be obtained after its resection. If it were possible to identify a prognostic factor before surgery, it could significantly change the surgical option.

The aim of this study was to determine whether there is a correlation between serum values of AP and LDH and the percentage of tumor necrosis in the extracted tumor after chemotherapy in patients with osteosarcoma.

Ambroszkiewicz et al.^18^ used reported values of AP, osteocalcin and C-terminal telopeptide as a prognostic factor for osteosarcoma in adolescents. Their conclusion was that patients with poor response to treatment showed elevated levels of serum AP. Bramer et al.^16^ studied 89 adult patients without reported metastasis. They concluded that patients who showed decreased serum value of AP after preoperative chemotherapy had a better survival prognosis. In the present study, however, we concluded that there is correlation between serum levels of AP and LDH pre- or post-chemotherapy and the percentage of reported tumor necrosis according to the Huvos index^12^, indicating that there is also no correlation between serum values of the enzymes and prognosis for patients.

In a study by Gonzáles-Billalabeitia et al.^19^ the authors evaluated 66 patients with osteosarcoma from June 1977 to March 2003. They used LDH serum levels as a prognostic factor for global survival rate of patients with osteosarcoma. Only the results of the preoperative chemotherapy were used, though. The authors concluded that patients with elevated serum levels of LDH had a worse survival prognosis when compared to patients with normal serum levels.

In another study, Lopez-Aguilar et al.,^20^ used LDH serum levels as a prognostic factor for lung metastases in patients with osteosarcoma. This study included 18 patients with normal and elevated values of LDH. The conclusion was that the serum value of LDH used as one factor is not related to the prognosis for patients.

Limmahakhun et al.^21^ published a study in which they analyzed the importance of AP and LDH as prognostic factors for progression of osteosarcoma. unhealthy and healthy patients were enrolled. Unlike our study, this study only used pre-chemotherapy serum values. They concluded that this serum marker was not relevant for the prognosis of osteosarcoma progression.

Adamcová-Krakorova et al.^22^ published a study with 36 patients with osteosarcoma, of which 70% had a poor response to chemotherapy. They reported that the life expectancy of these patients was 23 months. As part of monitoring, they measured the serum levels of AP and LDH. They reported that serum levels of AP and LDH were not considered prognostic factors for the percentage of tumor necrosis nor for survival, as well as the results of the present study.

Two studies published by Meyers et al.,^23^ ^,^ ^24^ prognostic factors such as age, gender, tumor location, the response to chemotherapy and serum levels of AP and LDH were analyzed in patients with osteosarcoma, including patients with metastases. The response to chemotherapy and serum values of AP and LDH were good predictors for survival prognosis. However, the authors did not study the relationship between serum values ​​of AP and LDH and the percentage of tumor necrosis, which was the main objective of our study. They also reported that patients with high AP and LDH levels had decreased survival rates as compared to patients with normal values.

Finally, we believe that this is the only study that tested the correlation between serum values of AP and LDH pre- and post-chemotherapy with the percentage of tumor necrosis, observing no relationship between them.

## CONCLUSION

Serum levels of AP and LDH show no correlation with the percentage of tumor necrosis in patients with osteosarcoma.
